# Emissions of VOCs From Polymer-Based Consumer Products: From Emission Data of Real Samples to the Assessment of Inhalation Exposure

**DOI:** 10.3389/fpubh.2019.00202

**Published:** 2019-08-14

**Authors:** Morgane Even, Mathilde Girard, Anna Rich, Christoph Hutzler, Andreas Luch

**Affiliations:** Department of Chemical and Product Safety, German Federal Institute for Risk Assessment (BfR), Berlin, Germany

**Keywords:** emission, VOCs, toys, polymers, consumer, exposure

## Abstract

The development of consumerism led to an increase in toy production. Such consumer products may contain non-intentionally added toxic substances that can emit from the product and may be inhaled by the consumer. Little data is available on the inhalation exposure of humans to volatile organic compounds (VOCs) from consumer products, so a reliable exposure assessment is needed. Only the emission chamber technique developed for building material emissions can provide solid estimations as it allows the products to be studied under real room conditions. This paper proposes a strategy to interpret emission experiment results from consumer products and assess the corresponding potential risk. It focuses on 14 common VOCs. The identification of the polymer type of 41 plastic articles was first performed by pyrolysis coupled online to gas chromatography with mass spectrometric detection (pyr-GC/MS). Their VOC profile was also determined by Dynamic Headspace-GC/MS (DHS-GC/MS). Softer polymers caused higher and broader emission profiles. Four specific toy samples were selected to be studied in a 203 l emission chamber and their emissions were compared to a reference material. A rapid decrease in the emissions was observed for each product and VOC. Based on these emission curves over time, the corresponding indoor air concentration could be calculated for the target VOCs for short-term or long-term exposures. The indoor air levels obtained were at least 35 times lower than the levels according to conventional indoor air guidelines. Guideline values were only exceeded using very conservative exposure scenarios.

## Introduction

Today, we are living in a globalized world, with an economy that facilitates the global exchange of goods and people. The development of easier transportation means led to the establishment of multinational companies that export goods worldwide. The total revenue of the global toy market has significantly increased over the past decade (1). This competitiveness prompts manufacturers to produce more attractive and cheaper products, sometimes at the expense of quality. Moreover, it becomes more and more difficult to control goods entering the national market, as a significant part of the products is ordered directly by the client via the internet from one country to another.

Consumer products can contain harmful substances, such as residual solvents, monomers, or additives (2). Consumer complaints about strong and unpleasant odors from toys were registered and several studies addressed the identification of off-odorants in toys (3–5). These odors are linked to emissions of volatile organic compounds (VOCs) from the products that may also release other non-odorous but harmful substances. The emitted substances will end up in the indoor air and may negatively influence the occupants' health. Indoor air quality is a growing concern as humans are nowadays spending 65% of their time at home (6) and also staying indoors when they work or commute. The air change rates are decreasing because of enhanced insulation techniques (7). The overall exposure control can often be instituted by small individual actions (8) as the most important sources of pollution are small and close to the person (9). Main sources of short term emission of VOCs indoors are personal care and cleaning products or cooking. Furniture and building material emissions (10) are considered as important emission sources too as they result in long term emissions.

The emissions of VOCs from building materials have been studied in detail for some time (11). This resulted in the development of standardized analytical methods and health-related indoor air guidelines [AIR or LCI values (12, 13)]. However, consumer products (e.g., toys, water toys, decoration products) may also emit harmful substances that can be inhaled by the occupants. So far, the results on the evaluation of inhalation exposure due to consumer products are insufficient for an adequate exposure assessment, which is required for reliable risk assessment. Much work on the emissions of VOCs from similar products has been carried out with headspace techniques, with a syringe (14), Solid-Phase-Micro-Extraction-GC/MS (SPME-GC/MS) (15) or DHS-GC/MS (16). While such experiments provide results on emitted compounds, they give no claim regarding possible risks from product use (14). In fact, it does not allow the description of emission kinetics under consumer-relevant conditions and a realistic exposure assessment. No standard method exists to determine the inhalation exposure to consumer products. There is a need for more data to provide a realistic evaluation of the inhalation exposure of consumers.

The emission test chamber method (17) can be used for this purpose. It was developed to determine the emission of VOCs from building products and furniture under indoor air conditions. Air samples can be collected on sorbent materials and analyzed via GC/MS (18). This method has already been used for such products: The emissions of allergenic fragrances from scented toys were studied in a 1,000 l emission chamber over 28 days (19) while the emissions of VOCs from “squishy toys” were characterized in a 113 l emission chamber (20) after 1 h or 3 days. The present work focuses on pre-selected non-scented products and non-intentionally added substances (NIAS) and presents 28 day emission profiles and a varied range of samples while describing a method for exposure assessment.

This work focuses on 14 substances which are summarized in Table 1. These compounds depict a broad range of physico-chemical properties (volatility, molecular weight, or polarity) and were all found in toy or consumer product samples by the German enforcement laboratories. Most of them are also described in the literature (3, 5, 16, 20) and some of them like benzene have carcinogenic, mutagenic, or reprotoxic properties (22) (Table 1). To evaluate the viability of reference materials to depict reliable emission profiles, a polyurethane material was spiked with the 14 substances in order to compare their emission profiles to real samples.

**Table 1:** Target VOCs with associated CAS number, boiling points (T_B_), molecular weights (MW), octanol-water partition coefficient (LogPow) (21), carcinogenic, mutagenic, reprotoxic (CMR) properties (22), and indoor air concentration guidelines (mg/m^3^) (12, 13, 23).

**Name**	**CAS**	**T_**B**_(**°**C)**	**MW**	**LogPow**	**C**	**M**	**R**	**AIR RWI**	**RWII**	**EU-LCI**	**NIK**	**DNEL**
Benzene	71-43-2	80	78	2.1	1A	1B	–	–	–	–	–	–
Toluene	108-88-3	111	92	2.7	–	–	2	0.3	3	2.9	1.9	192
m-Xylene	108-38-3	139	106	3.2								
p-Xylene	106-42-3	138	106	3.2	–	–	–	0.1	0.8	0.5	2.2	221
o-Xylene	95-47-6	144	106	3.1								
Dimethylformamide	68-12-2	153	73	−1.0	–	–	1B	–	–	–	0.015	15
Cyclohexanone	108-94-1	156	98	0.8	–	–	–	–	–	0.41	0.41	40
Phenol	108-95-2	182	94	1.5	–	2	–	0.02	0.2	–	0.01	8
Acetophenone	98-86-2	202	120	1.6	–	–	–	–	–	0.49	0.49	22
2–Phenyl−2–propanol	617-94-7	202	136	1.8	–	–	–	–	–	–	–	–
Formamide	75-12-7	210	45	−0.8	–	–	1B	–	–	–	–	6.6
Isophorone	78-59-1	215	138	1.6	2	–	–	–	–	–	–	11
Naphthalene	91-20-3	218	128	3.3	2	–	–	0.01	0.03	–	0.05	25
Dodecanol	112-53-8	259	186	5.1	–	–	–	–	–	–	–	–

As 28 day tests are costly and long, a sample pre-selection was carried out. First, the polymer structure of 41 plastic consumer products was characterized via Pyrolysis-GC/MS. In parallel, their VOC profile was determined via DHS-GC/MS. Four samples with high emissions of target compounds were then successively placed in the 203 l emission chamber. Air samples were taken regularly to follow the emission kinetics and therefore provide a complete estimation of the inhalation exposure over 28 days. The exposure was assessed by calculating indoor air concentrations and comparing them to indoor air guideline values (Table 1). The goal of this work was to introduce an effective strategy for sample selection and interpretation of emission testing results to provide solid estimations of the inhalation exposure to VOCs in consumer products.

## Materials and Methods

### Chemicals

The VOC ingredients under consideration are summarized in Table 1 and were supplied from Merck (Darmstadt, Germany) and Sigma-Aldrich (St. Louis, MO, USA). Ethyl acetate of analytical grade was obtained from Merck and used as organic solvent for all solutions.

### Materials

Standard material and real samples were studied. The standard plasticized polyurethane reference material plates were spiked with 12 different VOCs. The three xylene isomers were spiked as a single substance. Polyurethane was chosen because it can be synthesized at low temperature to prevent the loss of the VOCs spiked during the synthesis. It was doped at a target concentration of 1 mg/g, which enables the observation of all substances' emissions while still being realistic for material contamination, and custom synthesized by Polymaterials AG (Kaufbeuren, Germany). The plates had DIN A4 dimensions (21.0 × 29.7 cm) with a thickness of 6 ± 0.2 mm and a hardness of shore 70. Pieces of 12 × 10 cm were cut from the plate with a precision knife from NT cutter (Osaka, Japan) and placed in the emission chamber. Cutting metal utensils were cleaned twice with ethyl acetate and dried in the lab air before use. Until usage, the reference materials were kept at −18°C in gas-tight bags made of aluminum composite-layer film. Before starting the measurements, the pieces of reference material were allowed to adapt to room temperature and the bags were opened immediately before loading the chambers.

Forty-one plastic toys or decoration products were bought from local shops and kept in gas-tight bags made of aluminum composite-layer film at room concentration until usage. Four specific samples were used further for emission experiments, their properties are summarized in Table 2.

**Table 2:** Overview of the four selected samples for emission chamber experiments.

**No**	**Description**	**Dimensions per piece (cm^**3**^)**	**Manufacturer/importer**	**Country of origin**	**Identified polymer**
#1	2 water wings turtle	13.6 × 13.9 × 14.4	I	China	PVC
#2	Plopper animal figurine	13.8 × 7.8 × 8.8	II	China	PVC
#3	Plopper penguin figurine	14.6 × 8.5 × 8.1	II	Not given	PVC
#4	10 piece puzzle play mat	32.2 × 32.2 × 1.0	III	Not given	PE

### Pyr-GC/MS

Pyr-GC/MS enables the identification of the polymer structure via a thermal degradation of the polymer and the identification of the degradation products. A pyrolysis filament mounted on a Pyrolyzer Module was localized in a thermal desorption unit (TDU). It was connected to a cooled Injection System (CIS) equipped with liquid nitrogen cooling and a MultiPurpose Sampler (MPS2-XL; all items from Gerstel, Mülheim an der Ruhr, Germany). The CIS was installed into a gas chromatograph coupled with the mass selective detector 7890A-5975C GC/MSD System (Agilent, Santa Clara, CA). Pyrolysis of a polystyrene solution was performed regularly at 500°C for 0.33 min to assure a reliable performance of the system by evaluating the signal intensity for styrene oligomers. A constant ratio of the monomer-signal to the trimer-signal was used as quality requirement: a value of 3.9 ± 0.6 was indicative of an appropriate pyrolysis. Pieces of the polymers were cut as small as possible and placed directly into pyrolysis tubes (Gerstel) above a glass wool piece. A solvent vent method was applied: 60 ml/min helium (purity ≥99.999%, Linde, Pullach, Germany) passed into the Pyr-TDU-CIS complex using a pneumatic gas regulator (Gerstel) and a vent pressure of 30 kPa was applied. The pyrolysis took place subsequently at a temperature of 700°C to decompose the polymer (24). A lead time of 0.10 min, an initial time of 0.33 min and a follow-up time of 1.00 min were used. The TDU temperature was initially set at 50°C, followed by a ramp at 720°C/min up to 320°C and a final hold for 1.43 min. The CIS initial temperature was held at −120°C using cryo cooling to capture the volatile pyrolysis products for 4 min and was followed by a ramp of 12°C/min up to 320°C and a final hold for further 3.0 min.

Chromatographic separation was performed using an HP-5MS GC column (30 × 0.25 mm i.d., 0.25 μm df, Agilent) equipped with a pre-column (10 × 0.25 mm i.d., Phenomenex, Aschaffenburg, Germany). Helium gas (purity ≥99.999%) from Linde was used as a carrier gas at a constant flow of 1.0 ml/min. The GC oven was heated at 50°C for 6 min, ramped with 10°C/min to 320°C and held for an additional 3 min. MS analyses were carried out by collecting total ion chromatograms in the full scan mode and the mass range of 30–500 amu. Data were processed with the Chemstation E.02.02 software from Agilent. The degradation products were identified utilizing the NIST 11 mass library with the spectral search program version 2.0 distributed by Agilent.

### Dynamic Headspace

DHS was used to provide a quick determination of the VOC profile of the 41 samples. It does not lead to a direct exposure assessment, but the existence of an air exchange allows the comparison of samples under realistic conditions. Pieces of 0.13–3.03 g were cut with baked metal scissors to fill approximately half of 20 ml glass headspace vials tightly closed with magnetic silicon/PTFE caps. After a few hours of equilibration at room temperature, the headspace was purged with 600 ml nitrogen (purity ≥99.999%, Linde) with a flow rate of 15 ml/min. Analytes were trapped at 23°C on glass desorption tubes (6 × 0.4 cm i.d. × 0.6 cm o.d.) from Gerstel filled with Tenax TA®. The transfer heater between the vial and the desorption tube was held constant at 50°C. A conditioned desorption tube was used for each sample vial (procedure described in section Air Sampling From the Emission Chamber). The DHS apparatus was cleaned between each run: an empty glass vial was flushed with 1,000 ml at a flowrate of 100 ml/min at 70°C while the transfer heater was held at 70°C.

A DHS score was calculated for each target analyte and each sample. The signal area (A_sample_) was normalized with the IS (internal standard) signal area, corrected with the blank and normalized with the sample weight so that the results are comparable:

$$\begin{array}{l} \text{DHS score}=\frac{\frac{{\text{A}}_{\text{sample}}}{{\text{A}}_{\text{IS sample}}}\;-\frac{{\text{A}}_{\text{blank}}}{{\text{A}}_{\text{IS blank}}}}{\text{Sample weight}} \end{array}$$

### Emission Chamber

**Figure 1:**
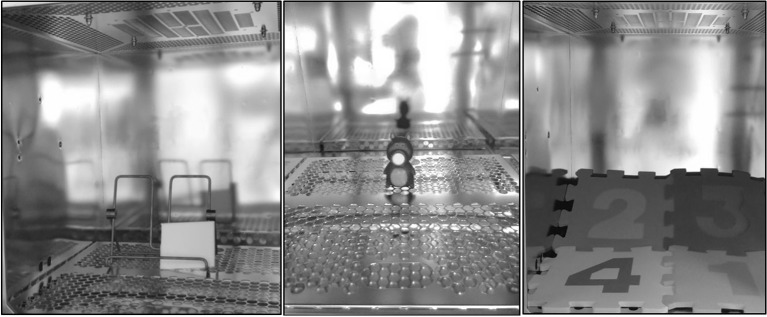
Placement of the standard polyurethane plate (left), sample #2 (middle), and sample #4 (right) in the emission chamber.

In experiments of this kind, the test sample is placed in an emission chamber which consists of a material with low adsorption and desorption characteristics. The environmental parameters in the chamber are regulated to mimic an indoor environment. A 203 l VOC emission test chamber model VCE 200 from Vötsch Industrietechnik (Balingen-Frommern, Germany) was used for emission testing. It had an inner chamber made of electro-polished stainless steel and a ventilator to ensure homogeneous air distribution. The standard plate pieces were placed on an easel while the toy samples were placed on the metal grid positioned at the bottom of the chamber (see Figure 1). Four samples with high emissions were pre-selected: The whole figurine for sample #2 and #3, one water wing for sample #1 and four puzzle pieces for sample #4 were loaded in the chamber. The parameters were set up in line with ISO 16000-9 (17) to a temperature of 23 ± 2°C, 50 ± 5% relative humidity and an air change rate of 0.5/h.

### Air Sampling From the Emission Chamber

Active air sampling was performed following ISO 16000-6 (18) using glass desorption tubes (6 × 0.4 cm i.d. × 0.6 cm o.d.) from Gerstel filled with Tenax TA®. Active sampling of 500 or 600 ml was carried out using a Gillian Dual Mode Low Flow Sample pump (Sensidyne, FL, USA) with an airflow of 100 ml/min. Blank samples were taken before the emission chamber was loaded to ensure low blank values of the chamber. Different air samples were regularly collected over 28 days after sample loading. For the reference plate, the whole experiment was repeated while for the toys, two air samples were collected successively for each time point. Prior to sampling, tubes were conditioned over 3 h with a nitrogen flow of 75 ml/min at 300°C. p-Xylol-d_10_ at 1 ng/μl or cyclodecane at 50 or 500 ng/μl in ethyl acetate were used as internal standard solutions in the different tests and stored in a freezer (−18°C). One microliter of internal standard solution was manually spiked with a rinsed 1 μl microvolume syringe (Trajan, Victoria, Australia) onto the desorption tubes. It was dried with 100 ml lab air or nitrogen at a flow rate of 100 ml/min. The tubes were stored in tight plastic storage containers from Gerstel at room temperature and subsequently loaded and analyzed within 1 week.

### Analysis of Air Samples (DHS and Emission Chamber)

The analysis was also carried out following ISO 16000-6 (18). Thermal desorption was performed in a TDU from Gerstel that had been connected to an Agilent 6890 gas chromatograph coupled with an Agilent 5975 mass selective detector. A helium gas flow and the following temperature program were used: 25°C for 0.2 min, then increase at 700°C/min to 280°C and final hold for further 2 min. During thermal desorption, analytes were cryotrapped with liquid nitrogen from Linde at −150°C in the CIS from Gerstel equipped with a liner filled with deactivated glass wool. After desorption, the CIS was heated up to 285°C at 12°C/s and then held for 15 min. Analysis from air samples was adapted from splitless to split 1:400 to allow every analyte to be quantified in its linear range.

The gas chromatograph (GC) was equipped with a DB-5MS column (60 m × 0.32 mm i.d., 1.00 μm df) (J & W Scientific, Folsom, CA, USA). Helium gas (purity ≥99.999%) from Linde was used as a carrier gas at a constant flow of 1.4 ml/min. The GC oven temperature started at 45°C for 0.5 min, was heated up to 200°C at 12°C/min, held for 5 min then heated up to 280°C at 20°C/min and held for 10 min.

The temperatures of the transfer line, quadrupole, and ion source were 295, 150, and 230°C, respectively. The mass spectrometer was used in combined SIM-Scan mode. The mass range in full scan was 40–450 m/z with a scan rate of 3.5/s. The target compounds were identified by comparison of their retention times and mass spectra with those of authentic standards. Quantification was done with SIM data for each target compound and internal standard. One quantifier and one or two qualifier ions were used (see Table 3), with dwell times of 10 ms.

**Table 3:** Analytical parameters: Retention time (RT), quantifier and qualifier ions.

**Name**	**RT (min)**	**Quantifier**	**Qualifier 1**	**Qualifier 2**
Benzene	6.5	78	52	77
Toluene	8.0	91	92	–
m/p-Xylene	9.7	91	106	–
o-Xylene	10.2	91	106	–
Dimethylformamide	8.1	73	44	–
Cyclohexanone	10.2	55	98	69
Phenol	11.1	94	66	65
Acetophenone	12.8	105	77	120
2–Phenyl−2–propanol	13.0	121	77	43
Formamide	6.8	45	44	43
Isophorone	13.6	82	138	–
Naphthalene	14.8	128	127	102
Dodecanol	19.3	55	69	83

### Quantitative Analysis of VOC Emissions

Analytes were quantified by internal calibration. Cleaned desorption tubes were first spiked with internal standards as described in section Air Sampling From the Emission Chamber. One microliter of the prepared solution in ethyl acetate, stored in a freezer (−18°C), was then also spiked with a rinsed 1 μl microvolume syringe (Trajan).The tubes were then dried, depending on the sampling volume, with 500 or 600 ml lab air or nitrogen at 100 ml/min. Subsequently, desorption tubes for calibration were analyzed as described in section Analysis of Air Samples (DHS and Emission Chamber).

Data processing was performed with the Mass Hunter Quant Software (B.05.00) from Agilent. The results were calculated as piece-specific emission rate according to the ISO (International Organization for Standardization) norm 16000-9 (17) and extrapolated for sample #1 and #4 to the whole sample:

$$\begin{array}{l} \text{SERpiece}=\frac{\text{Cchamber}*\text{Vchamber}*\text{n}}{\text{F}} \end{array}$$

with SER_piece_: Piece-specific emission rate (μg/h)
C_chamber_: Analyte concentration in the chamber (μg/m^3^)
V_chamber_: Volume of the chamber (m^3^)
n: Air change rate (/h)
F: Fraction of the whole sample placed in the chamber

The dispersion of values is displayed with the standard deviation of two measurements.

### Exposure Assessment

The room concentration is calculated for a 30 m^3^ room according to EN 16516:2017 (24) and compared to indoor air guideline values. The German Committee on Indoor Guide Values derives limit values (AIR-values, RWI, and RWII) that are based on current toxicological and epidemiological knowledge and correspond to levels where no health impairment is expected, even with a life-long exposition (12). The European LCI (lowest concentration of interest) values or the corresponding German NIK values (13) are usually compared to the emissions of building materials determined after 28 days of testing in the emission chamber. Unlike building materials, toys may be used directly after unpacking, so their emissions for the first hours were here compared to EU-LCI guideline values. The DNEL (Derived No-Effect Level) (23) values are defined by the German social accident insurance as derived exposure levels above which workers should not be exposed. The different values for the target analytes are shown in Table 1. AIR and LCI/NIK values are therefore chronic values while the DNEL is an acute level. For consumer products, lower DNEL values should be considered as they can affect more sensible groups (e.g., children), but those values were not available.

## Results

### Material Characterization

To characterize the polymer structure, chromatograms obtained upon pyrolysis of the consumer product samples were analyzed. The degradation products were identified and the peak list of each sample was compared to model chromatograms found in reference literature (25). Among 41 samples, 26 were identified as polyvinylchloride (PVC), 4 as polyethylene (PE), 4 as methyl methacrylate-butadiene-styrene copolymer, 3 as styrene-butadiene-styrene copolymer, 2 as polystyrene, 1 as polydimethylsiloxane, and 1 as polypropylene. The regular pattern of alkene chains surrounded by their corresponding diene and alkane analogous compounds is for example characteristic of polyethylene degradation. In all PVC samples, the presence of halogen could be confirmed via the Beilstein test: when the samples were brought in contact with copper, an applied flame turned green.

### VOC Profile

The VOC profile was characterized by application of DHS-GC/MS to all 41 samples.

$$\begin{array}{l} \text{DHS score}=\frac{\frac{{\text{A}}_{\text{sample}}}{{\text{A}}_{\text{IS sample}}}\;-\frac{{\text{A}}_{\text{blank}}}{{\text{A}}_{\text{IS blank}}}}{\text{Sample weight}} \end{array}$$

**Figure 2:**
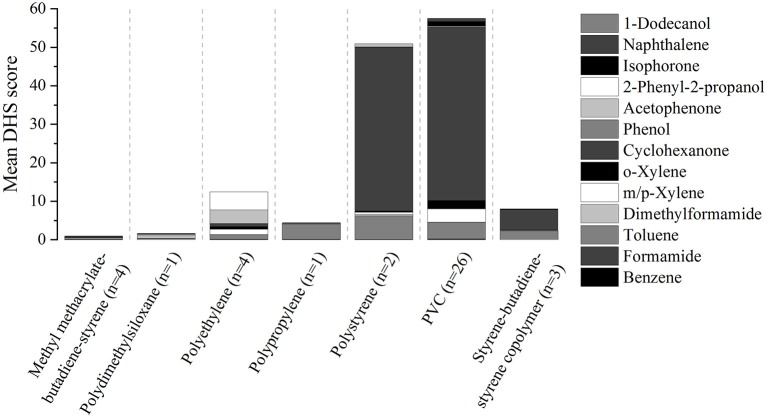
Sum of mean analyte DHS score for each type of polymer.

In Figure 2, the sum of the mean analyte DHS score is depicted for each polymer type. The highest scores were found for cyclohexanone, toluene and 2-phenyl-2-propanol. It could be observed that softer plastics like PVC, polystyrene or PE emit larger quantities compared to hard ones like polydimethylsiloxane or methyl methacrylate-butadiene-styrene copolymer. This was probably due to the polymer structure: More porous polymer may contain and emit more VOCs because of more inner free space and easier diffusion processes.

Three PVC samples and one PE sample were selected for subsequent emission testing (Table 2) because they led to relative high scores of different target analytes and the remaining sample quantity was representative and big enough for the emission chamber. An example for Pyr-GC/MS and DHS-GC/MS chromatograms is shown in Supplementary Figure 1 for sample #4.

### Quantification of Emissions

**Figure 3:**
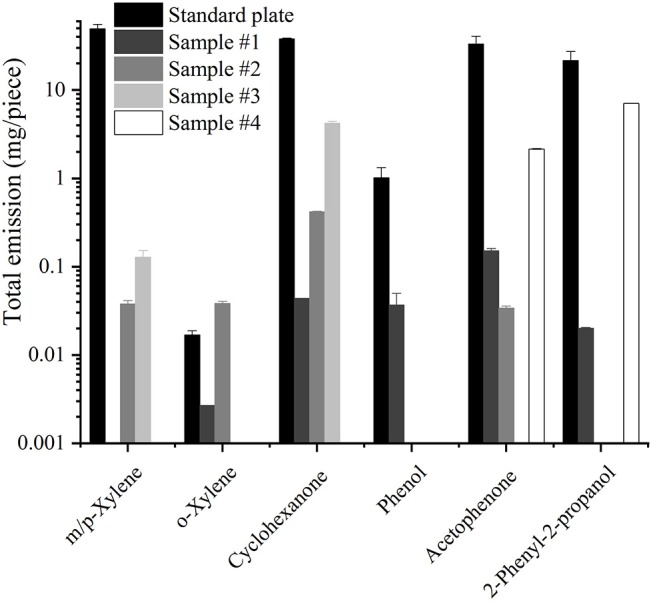
Total emission values during 28 days for 6 substances from the reference plate and the toy samples.

A piece of the reference material plate and four real samples were studied in the 203 l emission chamber for 28 days each. Six target analytes (m- or p-xylene were considered as one analyte as they cannot be separated completely by the chromatographic method used) could be detected in the toys' emissions. The total emitted amounts per toy or material plate, calculated by taking the area under the emission curve, are displayed in Figure 3 using a logarithmic scale. The standard reference plate spiked with 1 mg/g emitted much higher quantities compared to the four real samples. The most similar emission value was found for 2-phenyl-2-propanol from sample #4 with values only three times lower than from the polyurethane reference material. o-Xylene from the reference material showed much lower total emission values compared to the other target substances because only one isomer (m- or p-xylene) was spiked with 1 mg/g while the two others were impurities of the spiked substance.

**Figure 4:**
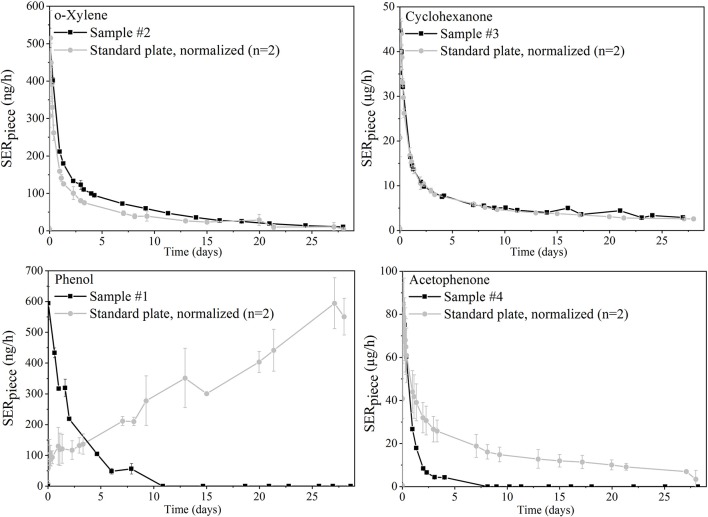
Emission profiles of four selected analytes from toy samples and comparison to normalized curves of the polyurethane plate (same maximum concentration).

The piece-specific emission rate (SER_piece_) profiles of four analytes emitted from the toy samples are shown in black on Figure 4. In gray, the curves obtained for the same analytes from the standard polyurethane plate were normalized to result in the same maximal emission rate.

### Exposure Assessment

**Figure 5:**
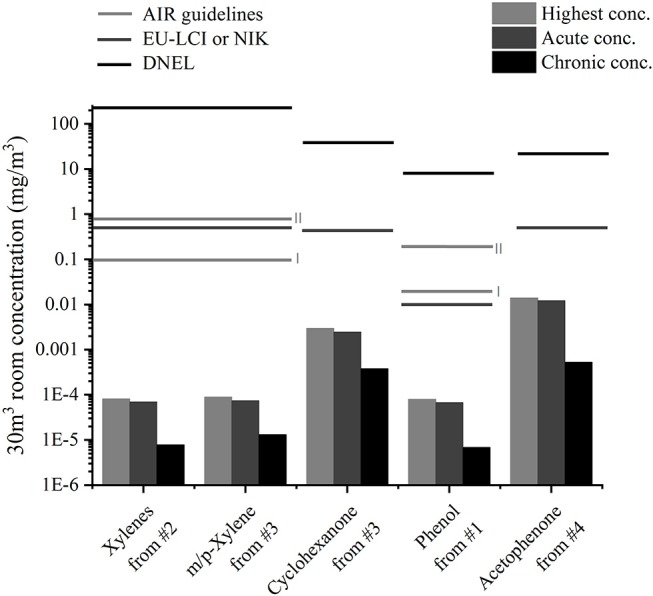
Calculated highest, acute (mean over first hours) and chronic (mean over 28 days) room concentrations of analytes from the four samples and comparison to indoor air concentration guideline values (12, 13, 23).

VOC concentrations found in the 203 l emission chambers can be diluted to look into the influence of the sample emissions on the room concentrations. The new European standard (26) suggests 30 m^3^ as the volume for a reference room. In Figure 5, the calculated room concentration of analytes for which indoor air concentration guidelines exist are depicted for each toy sample on a logarithmic scale and compared to guidelines. No indoor air guideline values exist for substances such as 2-phenyl-2-propanol, which was emitted at high concentrations from the play mat. The monitoring of VOC emissions over a month enables the study of different exposure cases: The maximum detected concentration (always after a few hours), the mean concentration in the first hours (from 7.1 for sample #3 to 14.9 h for sample #1 depending on sampling points) and the mean concentration over 28 days are shown. They, respectively, correspond to a peak exposure (child shortly playing at maximum concentration), a short-term exposure (child playing for a few hours or sleeping with the toy) and a long-term exposure (the toy stays a month in the child's room). Room concentrations induced by single samples were much lower compared to guideline values. The closest one was the maximum acetophenone concentration from a play mat which was 35 times lower than the EU-LCI value mentioned in (13).

## Discussion

### Interpretation of the DHS Experiments

For the DHS experiments, only a score based on the sum of the target substances was presented in the result part. Data were analyzed statistically to establish whether or not the polymer type correlates with the emission profile. For this purpose, it is possible to use Principal Component Analysis (PCA). A 3D plot of a PCA with three principle components was considered. No grouping based on the polymer structure could be determined when 29 target substances were considered.

### Emission Profiles

Figure 4 displays different emission profile shapes. They were compared between the standard plate and the toy samples for the different analytes of interest: For o-xylene from sample #3 and cyclohexanone from sample #2, the toy and plate profiles were very similar: The spiked reference material was in this case a good model to mimic real sample emission profiles.

However, the results can differ when the analyte depicts chemical properties that may interact with specific materials. Phenol emissions from the standard plate increased for example over 28 days while the phenol emission curve from sample #1 decreased. The synthesis of the polyurethane plates was conducted by reaction of an isocyanate and a polyol. It might be a reasonable explanation that part of the isocyanate functions also bound to the hydroxyl groups of the available VOCs. They were therefore released much more slowly than the other substances with the same volatility. Differences in emission profiles may also come from specific properties of the material itself. Acetophenone emissions decreased for example much more quickly from the play mat compared to the polyurethane standard material. This was probably caused by the fact that the play mat is made of foam which allows much faster internal diffusion compared to a polyurethane plate.

For all four toy samples, a quick decrease of the VOC emissions is observed in the first hours or days after unpacking. If we only take these compounds into account, a good advice would be to let the toy outside for a few days before use. However, other chemicals like semi-volatile organic compounds are not considered here and could result in persistent high exposure over longer time frames.

### Exposure Scenarios

**Figure 6:**
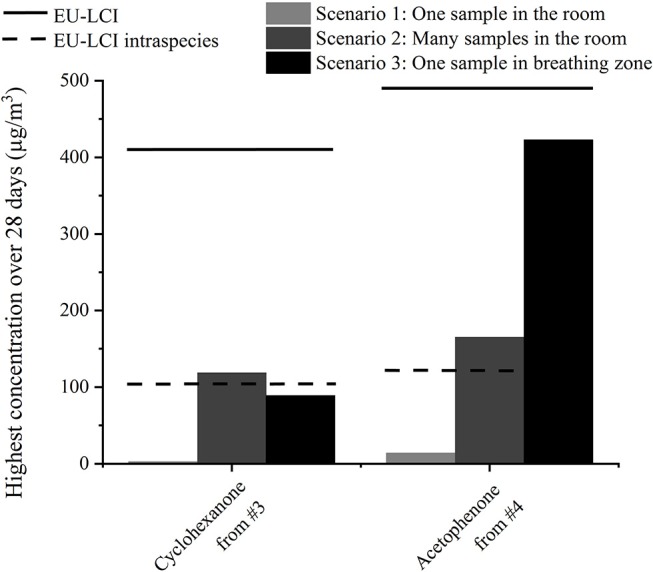
Calculated highest concentrations of two substances from two samples depending on the exposure scenario and comparison to the EU-LCI guidelines (13).

First assessments led to acceptable values for single substances from one product, but the realistic exposure scenario is more complex. Products often emit mixtures of substances that may trigger the same toxicological end point and therefore induce a cumulative effect. Moreover, children's toys only represent an additional source to human activities such as cooking, and other material emissions from building materials or furniture. Furthermore, other exposure scenarios different from the one used in section Exposure Assessment which considers only one sample in one 30 m^3^ room could be taken into account. In Figure 6, the highest emitted concentrations for two other scenarios are shown for cyclohexanone from sample #3 and acetophenone from sample #4. In the second scenario, not only one but several samples are present in the 30 m^3^ room: The floor of the model room (12 m^2^) is carpeted with the play mat or 40 figurines [as suggested in (20)] are present in the room. As the air distribution and ventilation may not be as good in a real room compared to the emission chamber, the air volume around the object may contain higher emission concentrations compared to the rest of the room. In the third scenario, a breathing zone of 1 m^3^ around the sample, as used in Masuck et al. (19), was considered. Those results were compared to the EU-LCI guidelines. As described in (20), an intra-species factor of four can be considered for a child, as it is more vulnerable than an adult. Only with this consideration did the emission concentrations exceed the guidelines for cyclohexanone from #3 in the first scenario and for acetophenone from #4 in both scenarios.

## Conclusion

This study was carried out to fill the data gap necessary for the exposure assessment of VOC emissions from consumer products made of polymers. It was first shown, based on 41 samples and DHS experiments, that softer polymers emit broader and higher VOC profiles than harder plastics. Four specific samples were selected to be studied in an emission chamber over 28 days and their emissions compared to those of a reference polyurethane plate spiked with 14 VOCs. The real samples led to lower total emitted quantities than the reference material spiked at 1 mg/g, but the emission curves could result in similar shapes depending on the analyte and material properties. The room concentrations derived from the emissions of each single sample in a 30 m^3^ room were lower than existing indoor air guidelines. However, when other scenarios with more samples in the room or a smaller breathing zone were considered, the highest emitted concentrations exceeded the EU-LCI guideline corrected with an intra-species factor for children. Those first estimations are of low concern as the EU-LCI is a guideline for chronic concentrations and it was only exceeded by the peak concentrations in extreme scenarios. It was demonstrated that the experimental approaches used and the strategy for calculating exposure scenarios for VOCs emitted from consumer products is feasible for risk assessment. To our best knowledge, this is the first study to give a full characterization of the polymer type and the VOC profile of consumer product samples as well as complete emission curves from reference materials or real samples and results on the inhalation exposure assessment. It should always be considered that consumer products may represent an additional source of VOC emissions beside well-known sources, such as building products. More data regarding emissions from consumer products will be necessary in the future to provide a better overview on the current market and make it possible to exclude health risks for consumers. Standardized methods will also be required to support the routing work of official control labs.

## Data Availability

The datasets generated for this study are available on request to the corresponding author.

## Author Contributions

Each author has participated in the work intellectually or practically and takes responsibility for the content of this article. ME, MG, and AR carried out the practical work and data analysis. ME, CH, and AL designed the study, interpreted the results, and contributed to the manuscript. The final version was approved by all authors.

### Conflict of Interest Statement

The authors declare that the research was conducted in the absence of any commercial or financial relationships that could be construed as a potential conflict of interest.
