# Supplementary material for: Emissions of VOCs From Polymer-Based Consumer Products: From Emission Data of Real Samples to the Assessment of Inhalation Exposure
Source: Front Public Health. 2019 Aug 14;7:202. doi: 10.3389/fpubh.2019.00202 (PMC6707103; doi:10.3389/fpubh.2019.00202)
Supplement: Supplementary file 1 [file Data_Sheet_1.docx]

Supplementary Material


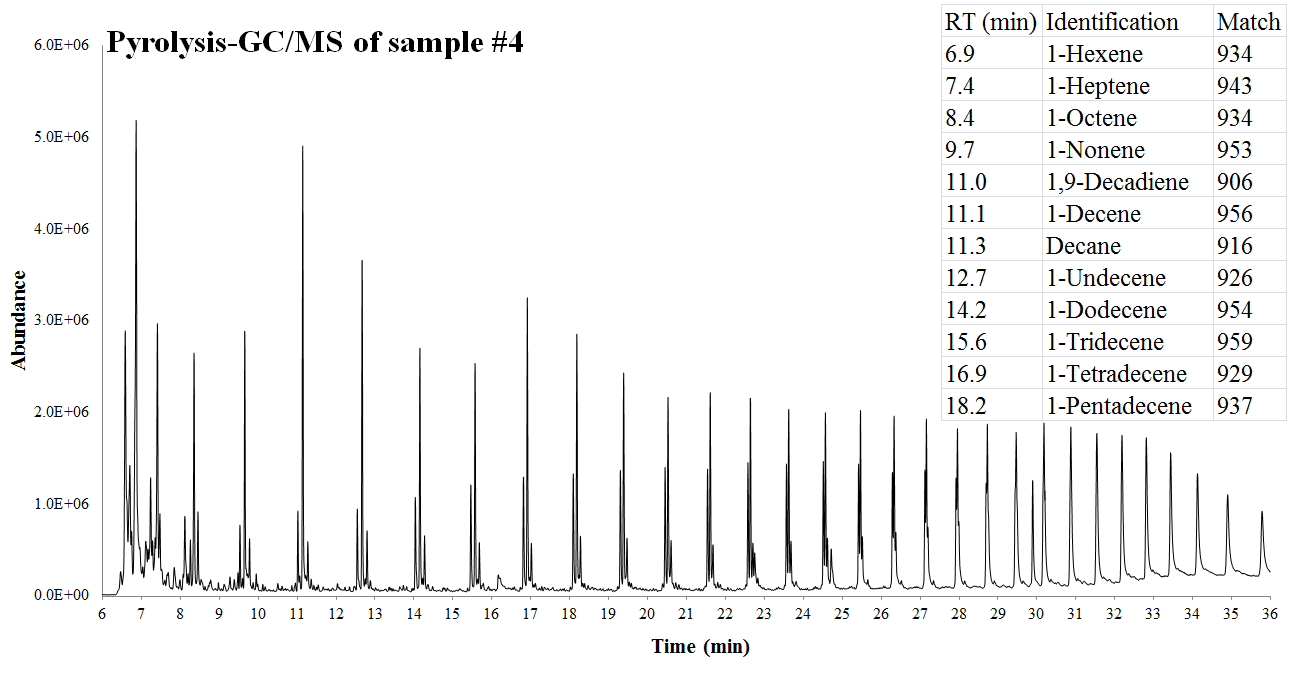


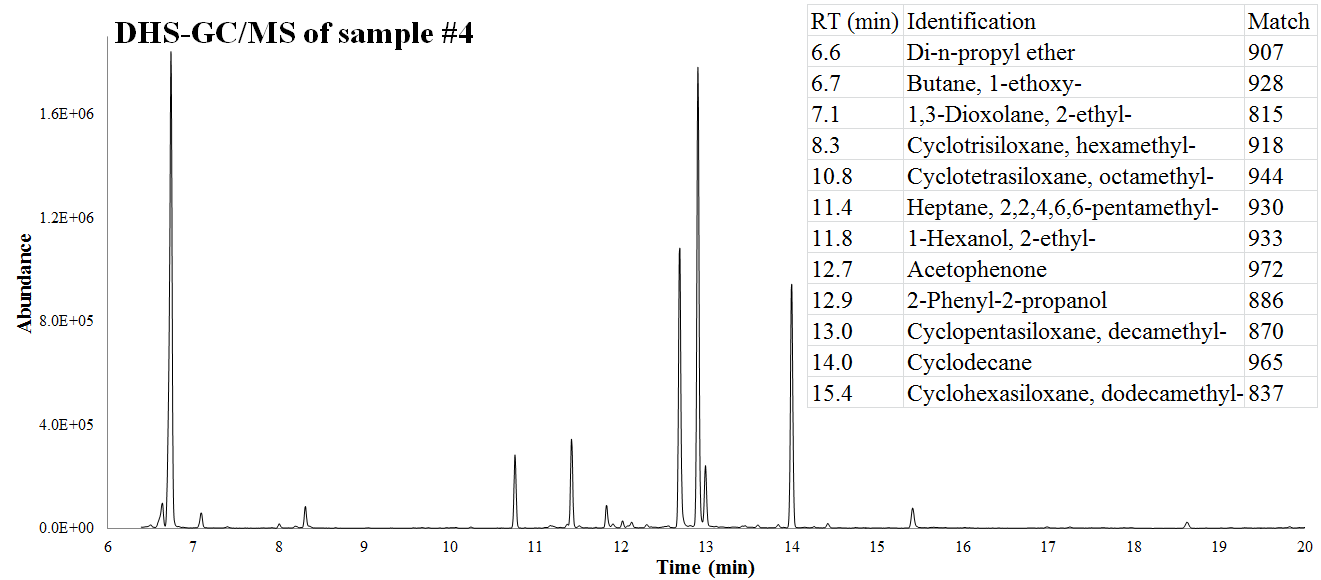


**Supplementary Figure 1.** GC/MS chromatograms in full scan mode of sample #4. Above: degradation products after pyrolysis at 700°C; below: emitted substances during DHS experiments at 23°C and 15 mL/min. Retention time (RT), NIST library identification and match score (maximum value 1000).
